# Diffuse Correlation Spectroscopy (DCS) for Assessment of Tissue Blood Flow in Skeletal Muscle: Recent Progress

**DOI:** 10.4172/2161-0940.1000128

**Published:** 2013-11-30

**Authors:** Yu Shang, Katelyn Gurley, Guoqiang Yu

**Affiliations:** 1 Department of Biomedical Engineering, University of Kentucky, Lexington, KY 40506, USA; 2 Department of Neurological Surgery, University of Louisville, Louisville, KY 40202, USA

**Keywords:** Diffuse Correlation Spectroscopy (DCS), Skeletal muscle, Blood flow, Motion artifact, Gating algorithm, Peripheral Arterial Disease (PAD)

## Abstract

Near-infrared diffuse correlation spectroscopy (DCS) is an emerging technology for monitoring blood flow in various tissues. This article reviews the recent progress of DCS for the assessment of skeletal muscle blood flow, including the developments in technology allowing use during dynamic exercise and muscular electrical stimulation, the utilization for diagnosis of muscle vascular diseases, and the applications for evaluating treatment effects. The limitations of current DCS studies and future perspective are finally discussed.

## Overview

Skeletal muscles comprise approximately 40% of total body mass, facilitating posture and locomotion and utilizing up to 85% total cardiac output [1]. Many vascular diseases, such as peripheral arterial disease (PAD), cause a decline in skeletal muscle blood flow [2–4], impairing the delivery of oxygen and other nutrients to the tissue. Correspondingly, many therapies have attempted to improve microcirculation and oxygen consumption in muscles. Monitoring of skeletal muscle blood flow and oxygen consumption rate is essential to provide insight into muscle pathophysiology and may help determine diagnosis and efficacy of treatment.

Blood flow in skeletal muscle has been quantified by a number of noninvasive methods. Conventional venous occlusion plethysmography has been employed in muscle perfusion investigations for many years [5,6]. This method, however, can be used only in the static state, since the occlusions interrupt muscle blood flow. Arterial-spin-labeled MRI (ASL-MRI) [7] and positron emission tomography (PET) [8] are also capable of monitoring microvasculature blood flow, but these methods require expensive and cumbersome equipment not available to all clinics or laboratories. Furthermore, these technologies are highly sensitive to motion artifacts which distort signals from the exercising muscle [9].

Oxygen consumption rate (V̇O_2_) has been quantified by a variety of techniques. Spirometry measures whole-body V̇O_2_, but cannot provide information about local muscle tissues [10]. Blood sampling is an established technique, but is invasive and remains regional in scope as the samples are taken from major vessels connected to groups of muscles [11,12]. The 31-phosphorous magnetic resonance spectroscopy (31P-MRS) can quantify local muscle V̇O_2_ [13], but requires expensive and cumbersome equipment which limits use in exercise studies. Additionally, ^31^P-MRS measurements have poor temporal resolution and limited sensitivity, inhibiting the ability to quantify rapidly changing dynamics.

A novel technique known as near-infrared (NIR) diffuse correlation spectroscopy (DCS) has been recently developed that enables quantifying relative changes in microvascular blood flow (rBF). DCS uses coherent near-infrared light to penetrate deep tissues and measures speckle fluctuations of the diffuse light that are sensitive to the motions of red blood cells in tissues [3,14,15]. DCS provides a portable, noninvasive, and inexpensive alternative for microvascular blood flow measurements and has been validated against other standards, including power spectral Doppler ultrasound [16], Doppler ultrasound [17,18], laser Doppler flowmetry [19,20], Xenon computed tomography [21], fluorescent microsphere flow measurement [22], and ASL-MRI [23]. The DCS technology has been extensively introduced into various tissues, including brain [18,21,24–29], tumor [30–33] and skeletal muscle [2,3,23,34–42]. The applications of DCS in brain and tumor have been previously reviewed [43–46]. Interested readers are encouraged to read these publications for details. This review paper will focus on introducing some recent progress of DCS in the study of skeletal muscles.

## Principle of DCS Technology

Historically, the motion of red blood cells in superficial tissues (<1 mm) was quantified by detecting light fluctuations within a single speckle area on tissue surface with a single scattering theory [47–49]. In thick/deep tissues such as skeletal muscles, the photons experience multiple scatterings with varied pathlengths. DCS is a theory that accounts for multiple scatterings of photons and quantifies the motion of red blood cells (i.e., the blood flow) in thick/deep tissues [14,50–52].

The key components of a DCS flow-meter include a long-coherence length laser at NIR range, a single-photon-counting avalanche photodiode (APD) detector and an autocorrelator board. Other components, such as source/detector fibers, computer, and A/D board, are used to couple light in/out of tissue or control/record optical data. For tissue blood flow measurement, the laser placed on the tissue surface (e.g., skin) launches long-coherence NIR light into the tissue via a multiple-mode source fiber, and the light transported/ scattered through the tissue was collected by a single-mode (or a few-mode) detector fiber placed millimeters to centimeters away from the source fiber. The detected light is then delivered via the detector fiber to APD detector, where the count of photons per unit time (i.e., light intensity) is recorded. The temporal fluctuation of light intensity in a single speckle area of tissue collected by the detector fiber is associated with the motion of moving scatterers (primarily red blood cells in microvasculature) and can be quantified by calculating the decay of light intensity autocorrelation function using the autocorrelator. The electric field temporal autocorrelation function of light is determined from the normalized light intensity autocorrelation function, and it obeys the correlation diffusion equation in highly scattering media [14,15]. By fitting the electric field autocorrelation curve to an analytical solution of correlation diffusion equation and assuming a particular flow model, the blood flow index is yielded [19,34]. Brownian diffuse motion is found to be the best flow model to fit the DCS autocorrelation curve, and the blood flow index is represented by αD_B_ [19,52]. Here D_B_ is the effective diffusive coefficient and α is the ratio of light scattering events from moving red blood cells to total scatters. For human skeletal muscles, a semi-infinite geometry is often assumed to obtain the analytical solution of correlation diffusion equation [2,19,39,41]. Because the absolute value of blood flow index (αD_B_) is affected by many factors (e.g., the coupling between fiber and tissue surface) and varies across subjects, most of studies only reported the relative change in blood flow (rBF) during or post physiological manipulation as compared to the baseline (before physiological manipulation) [3,4,20,23,27, 28, 34, 35, 39, 53].

## Technology Development: Gating Algorithm and Absolute Measurement

Exercise is a commonly used protocol to evaluate the working capacity of skeletal muscle as well as its circulatory and metabolic functions. However, measurement of blood flow during exercise is a difficult task, as flow measurement is susceptible to muscle fiber motions during exercise. Previous study has demonstrated that the muscle fiber motions increase moving scattering in tissue, introducing artifacts into DCS measurements and leading to overestimation of blood flow changes during exercise [34]. To overcome this limitation, a novel algorithm was recently proposed to gate DCS data acquisition based on the muscle activity status [42]. Recordings from a dynamometer, such as hand/foot position, torque and velocity, were used to determine the muscle status (contraction vs. relaxation) during exercise. The DCS control program recorded data only at the time when muscle was relaxed.

**Figure 1:**
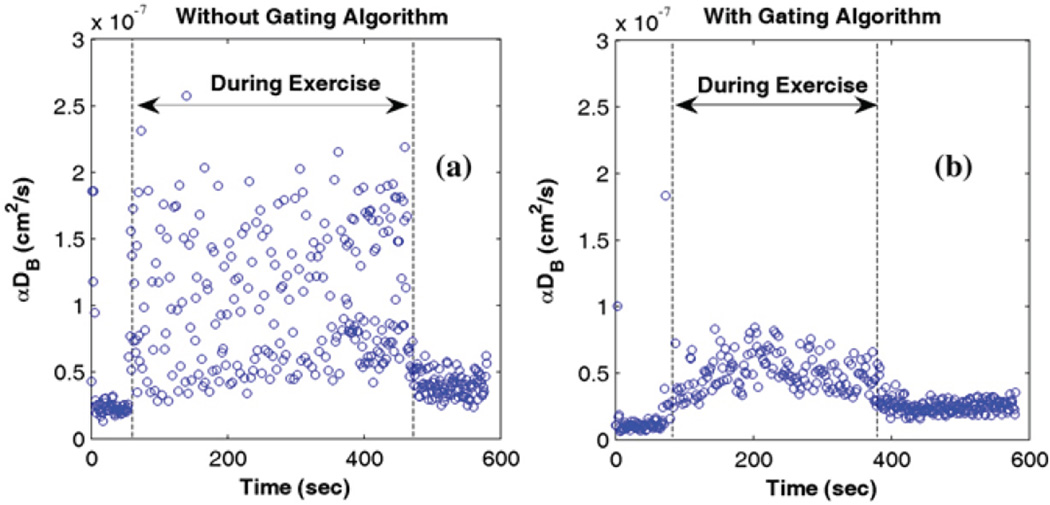
Blood flow index (αD_B_) measured by DCS during exercise without (a) and with (b) use of gating algorithm. The dashed vertical lines indicate the beginning and ending of exercise. Courtesy of K. Gurley [54].

This novel gating algorithm for minimizing the motion artifacts to blood flow measurements has been applied to a handgrip exercise study [42]. Figure 1 shows the blood flow index (i.e., αD_B_) measured by DCS during handgrip exercise without (Figure 1a) and with (Figure 1b) use of the gating algorithm, respectively. Apparently, muscle fiber motions during exercise introduced remarkable artifacts into DCS blood flow measurement (Figure 1a). The gating algorithm significantly reduced the motion artifacts and enabled recording of the true blood flow signals during exercise (Figure 1b).

**Figure 2:**
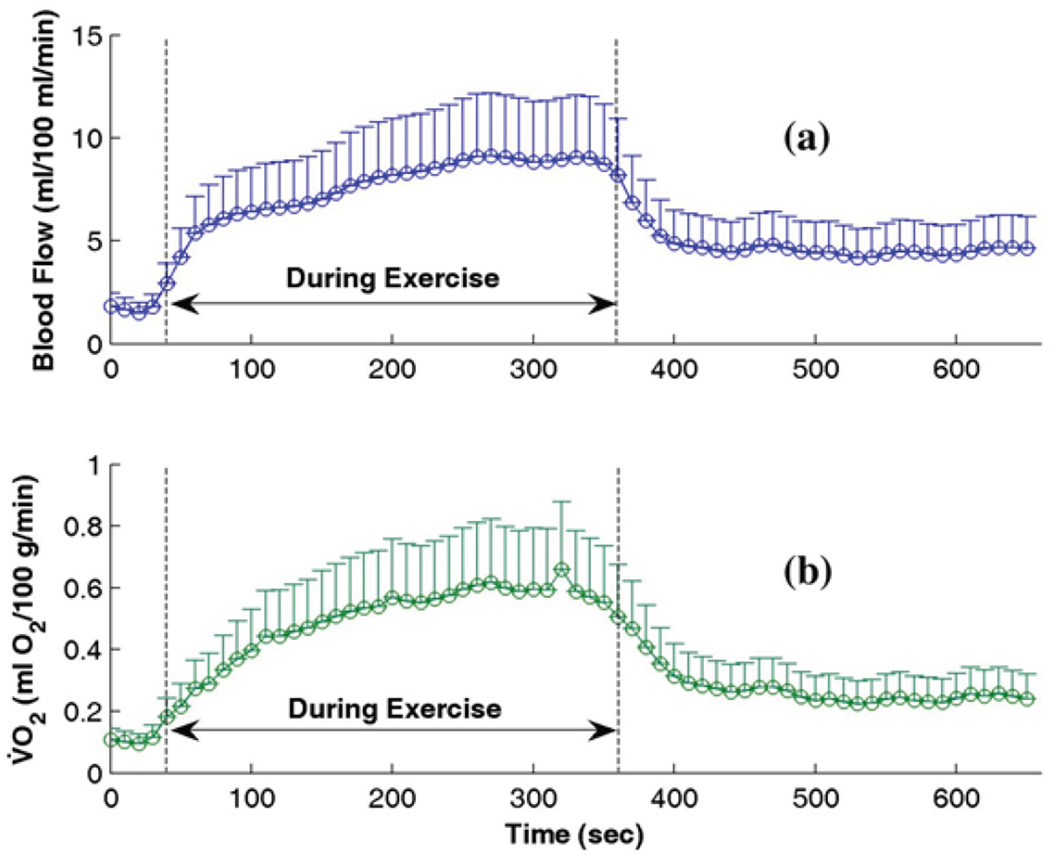
Time course data of absolute blood flow (a) and V̇O_2_ (b) in calf muscles (n = 9) measured by DCS/NIRS technologies throughout a 3-minute handgrip exercise. Each data point is presented as mean ± standard error over nine healthy subjects. The dashed vertical lines indicate the beginning and ending of exercise. Courtesy of K. Gurley [54].

DCS is a valuable tool providing reliable quantity of relative changes in blood flow (i.e., rBF) rather than its absolute value (αD_B_). To obtain an absolute measurement, a near-infrared spectroscopy (NIRS) oximeter was utilized in a calibration protocol to extract the absolute values of forearm blood flow and V̇O_2_ during a rest period (baseline) through venous and arterial occlusions of the upper arm [42,54]. Briefly, three venous occlusions at 50 mmHg and one arterial occlusion at 240 mmHg were applied sequentially on the upper arm of the subject. Forearm flexor muscle oxygenation data, including oxy-and deoxy-hemoglobin concentrations ([HbO_2_] and [Hb]), total hemoglobin concentration (THC), and blood oxygen saturation (S_t_O_2_), were acquired using a commercial NIRS oximeter (Imagent, ISS Inc., IL). Baseline blood flow was derived as the average rate of initial increase in THC during the first 5 seconds of three venous occlusions. Baseline V̇O_2_ was calculated as the linear regression of the oxygen desaturation rate (i.e., [HbO_2_]-[Hb]) during the first 60 seconds of arterial occlusion. The absolute values of blood flow and V̇O_2_ throughout (i.e., pre, during and post) exercise were calculated by multiplying the rBF and rV̇O_2_ and their absolute baseline values measured by the occlusion protocols, respectively. Here rV̇O_2_ represents the relative change of V̇O_2_ that was calculated from relative changes of blood flow and oxygenation data measured by the DCS/NIRS devices [3,39,42,54]. Figure 2 shows the time course data of absolute blood flow (Figure 2a) and V̇O_2_ (Figure 2b) in forearm muscles of nine healthy subjects throughout a 3-minute handgrip exercise. Data were recorded by the DCS/NIRS devices with the gating algorithm and calibration protocols. The baseline values of blood flow and V̇O_2_ as well as their dynamic changes throughout exercises were found to agree with previous literature [55–57].

Very recently, the novel gating algorithm was adapted in a muscular electrical stimulation (ES) study to facilitate blood flow and oxygenation measurements during ES on quadriceps muscle [58]. According to the gating algorithm, DCS/NIRS data were only recorded in muscle fiber relaxation status (determined by output current from the muscle stimulator). Measurement of muscle blood flow/oxygenation responses to ES offers an objective standard to optimize ES treatment for diseases caused by poor muscle blood circulation and oxygenation (e.g., pressure ulcer).

As an advanced application, DCS was used in another ES study to extract the strain rate of contracting or relaxing muscle fibers through fitting the correlation curve to a unique physical model [40]. Fast-and slow-twitch muscle fibers were differentiated with this advanced technique, indicating the potential of using DCS to provide deeper insight into muscle physiology.

## Diagnosis of Skeletal Muscle Diseases

DCS/NIRS has been used to assess muscle circulatory and metabolic function in diseased skeletal muscles [3,4,39]. A recent study investigated muscle rBF responses in healthy and PAD groups following treadmill and pedal exercises [4], showing significant differences between the two groups. Compared to the healthy subjects who exhibited elevation in muscle rBF after exercise, the PAD patients maintained a relatively stable muscle flow throughout exercise, implying some vascular deficit in responding to the metabolic demands of exercise.

DCS/NIRS was also used to quantify muscle hemodynamic and metabolic responses in older women with fibromyalgia following isometric pedal exercise [39]. Compared to the age-matched healthy controls, patients with fibromyalgia exhibited lower oxygen extraction rate although their rBF values did not show a significant difference.

## Evaluation of Surgical and Physical Treatment Effects

DCS technology was first applied in clinical surgery by monitoring muscle blood flow in patients with PAD during femoral arterial revascularization [2]. DCS demonstrated high sensitivity in detecting the instantaneous changes in rBF during surgical procedures as well as the rBF improvement immediately after revascularization. The acute improvements in calf muscle blood flow were associated with long-term significant improvements in symptoms and function. This pilot study corroborates potential of the diffuse optical methods for assessing the success of arterial revascularization.

In another application, DCS was used to evaluate the effect of massage on muscle microcirculation [38]. Immediate increase in calf muscle rBF was found following 8-min lower limb massage on young women, indicating the potential of DCS for assessing acute treatment effects of physical therapy.

## Study Limitations and Future Perspective

Currently, the blood flow index (αD_B_) in DCS technology is mostly calculated using the semi-infinite analytical solution [52,59,60], where the tissue is assumed to be homogenous. As a result, the yielding index reflects blood flow information not only from skeletal muscle, but also from skin and fat, as light also penetrates into these overlaying tissues. This phenomenon is well known as “partial volume effect” [27,28]. Realistically, blood flow responses from different tissues (skin, fat and muscle) differ remarkably [3], thus tissue heterogeneity should be taken into account. One of future goals is to overcome the limitation of this “partial volume effect” and precisely extract the blood flow in skeletal muscles by combining multiple source-detector separation data with the DCS solution for heterogeneous tissues. Furthermore, the approach to control DCS data acquisition during exercise may be improved for further reducing motion artifacts. The current gating algorithm utilizes extrinsic signals, such as foot position/velocity/torque or ES current, to determine the muscle status during exercise or ES. Intrinsic signals detected directly from the muscle (e.g., by electromyography) may be employed to precisely gate DCS measurements. With further technology developments and more clinical applications, DCS is expected to become the technique of choice for diagnosis and treatment management of muscular diseases.
